# Investigation of early literacy skills of preschool children with hearing loss

**DOI:** 10.1186/s13052-024-01578-0

**Published:** 2024-01-17

**Authors:** Pelin Pistav Akmese, Destina Sezgin Kucuk, Gulce Kirazli

**Affiliations:** 1 Audiology Department, Faculty of Health Sciences, Ege University, Izmir, Turkey; 2 Luden Ozel Kesif Special Education and Rehabilitation Center, Izmir, Turkey; 3 Audiology Department, Faculty of Health Sciences, Ege University, Izmir, Turkey

**Keywords:** Hearing loss, Hearing aid, Early language skills, Early literacy, Preschool children

## Abstract

**Background:**

Early literacy development is critical for children with hearing loss to develop literacy skills in the years to come. The aim of this study is to compare the early literacy skills of 60–72 months’ children with hearing loss to the results of children with normal hearing.

**Methods:**

A total of 40 children (20 children with hearing aid (HA) and 20 children with normal hearing (NH) were evaluated in the study. Receptive and expressive language was assessed by Test of Early Language Development (TELD-3) and Early Literacy Test (EROT) was applied to assess the early literacy skills of all children in the study.

**Results:**

The receptive and expressive language results of the hearing-impaired group were significantly lower than those of normal hearing. Moreover, in EROT when a general analysis is made with main test titles such as, the vocabulary knowledge, letter knowledge, the listening comprehension, results showed that there was a significant difference between the HA and NH groups.

**Conclusions:**

This study highlights the importance of supporting early literacy skills, which are prerequisite skills for reading and writing skills, in children who receive both mainstreaming education and special education in the risk group and/or continue their education in kindergarten.

## Background

Hearing is necessary for children to improve receptive and expressive language skills and to respond to social situations [6]. Congenital or pre-linguistic hearing loss (HL) can affect the child's language development and cause it to differentiate from normal hearing peers in cognitive, social and emotional development [8]. Even children with mild hearing loss have auditory memory difficulties, articulation impairment, and delay in the development of receptive and expressive language. As the degree of HL increases, children's speech production and vocabulary decrease,speech perception, literacy skills and academic success are reduced [29, 30]. Hearing loss, whether congenital or acquired during the prelingual phase, influences a child's language development, and causes delays in social, cognitive, literacy and emotional development as compared to peers.

Children who are deaf or hard of hearing are at a higher risk of severe language problems in the early stages of development and literacy difficulties later in life [1, 16, 23, 33]. Factors influencing the language and literacy development of children with hearing impairment,the degree of hearing loss is defined as a delay in language development, difficulties in developing vocabulary, difficulties in syntax, inadequacy of teachers and education programs, and inability to ensure family participation [5, 28].

Studies on the early literacy development of children with hearing impairment reveal that their literacy development goes through similar processes with their hearing peers [1, 7, 8, 25, 33]. Although it is stated that children with hearing impairment may develop slower in complex structures in language than their peers due to hearing impairment [10], through a rich literacy atmosphere, skilled adult–child interaction, and experiences acquired through child-centered approaches can display literate behaviours like their hearing peers [15, 34]. Moreover, there are few reports on early reading fundamental skills for young children who are deaf or hard of hearing [2, 10, 24].

The aim of this study is to compare the early literacy skills of children using hearing aids (HA) with the age between 60–72 months of preschool and/or special education with the results of children who have normal hearing (NH). The following questions were posed for this reason:

1. Is there any difference between the receptive and expressive language test results of children with HA and NH children?
2. Is there any difference between the early literacy test measurements of vocabulary knowledge, phonological awareness, letter knowledge and listening comprehension of children with HA and NH?
3. Do the mean scores of the receptive language, expressive language test and early literacy measurements such as vocabulary knowledge, phonological awareness, and letter knowledge and listening comprehension of HA and NH children, differ by gender?
4. Is there a significant relationship between the age, gender, receptive language, expressive language and EROT Scores; vocabulary knowledge, phonological awareness, and listening comprehension of the children with HA?
5. Which of the scores of vocabulary knowledge, phonological awareness, letter knowledge and listening comprehension predicted the HA group?

## Materials and methods

Volunteer children aged 5–6 (60–72 months) who applied to Ege University Faculty of Medicine, Department of Otorhinolaryngology after the approval of Ege University Medical ethics committee (Approval Date: 31.07.2018& Reference Number: 18–7.1/54) were included in the study. All methods were performed in accordance with the ethical standards in the Declaration of Helsinki. Written informed consent was obtained from the parents and/or legal guardians with ethical approval and consent. The sample size was determined as 40 (20 for each group) according to the power analysis calculation done using G*power 3.1 program, with an effect size of 0.95, a margin of error of 0.05, a confidence level of 0.90, and a population representation of 0.95. Therefore, 40 children who applied to Ege University's Department of Otorhinolaryngology and continued their kindergarten and/or special education were included. For the study group: 20 children aged 5–6 years who had sensorineural hearing loss and using hearing aids and were being followed for their routine controls at Ege University Faculty of Medicine, Department of Otorhinolaryngology. They did not have syndromic hearing loss, they only had idiopathic hearing loss; For the control group: 20 children admitted to Ege University Faculty of Medicine, Department of Otorhinolaryngology, no ear pathology was found as a result of the tests, and children aged 5–6 years with normal hearing were included. The research was conducted between May 2021 and December 2021.

Participants were summoned twice to the clinic. The participants were first tested for pure tone audiometry in the clinic during their first visit. The hearing aids were adjusted based on the test results. At their second visit, they were given language and early literacy tests.

The language and early literacy tests took approximately 40 min. 10-min breaks were given between tests according to the child's condition. The first group consists of 20 pre-school children who had hearing loss and using hearing aids. The second group consist of 20 children who have normal hearing matched for age and gender, volunteering to participate in the study. In all groups, the families of the children were filled with the ‘Demographic Form’, which includes demographic information prepared by the researchers, and the ‘Parental Consent Form’ indicating that the parents allowed the children to participate in the study, and then the children were administered an ‘Pure Tone Audiometry Test’, ‘Test of Early Language Development (TELD-3)’, ‘Early Literacy Test (EROT)’. The data were collected and analysed with appropriate statistical analysis methods using the SPSS 25.0 program.

### Demographic form

The demographic form is formed by the researchers, including questions about the children's families and children's demographic information. Information was obtained by means of individual interviews with the families who volunteered to participate in the study.

### Parental consent form

The parental consent form is formed by the researchers. Parents of each child were informed to participate in the language assessments to be carried out in the study. Parents were asked to read and sign the ‘Parental Consent.’

### Pure tone audiometry test

Before the pure tone audiometry test, an experienced audiologist examined the children's ears. An experienced audiologist used an inter acoustic (AC40) audiometer and supra-aural headphones in an acoustically shielded sound unit to perform the pure tone audiometry test. The pure tone audiometry test was conducted using conventional audiometry because all children were able to adapt to the test. Each ear's air and bone conduction pure tone threshold averages at 500, 1000, 2000, and 4000 Hz were calculated. As the average hearing threshold for each child, airway pure tone threshold averages were written separately for each ear.

Hearing thresholds obtained from the hearing tests of children in the HA group were recorded in the appropriate hearing aid adjustment program. Real-ear measurements were used to make adjustments based on The Desired Sensation Level (DSL) Version 5.0 target gains.

### Test of early language development-third edition (TELD-3)

The Turkish adaptation of the " (TELD-3)" developed by Guven and Topbas to determine children's receptive and expressive verbal language skills in the study. Language Development Test’s norm-based language test was used. TEDIL (TELD-3) has been adapted to Turkish to measure the early verbal language development of children between the ages of 2–0 and 7–11, whose native language is Turkish. It consists of two parallel forms, A and B form. It includes two subtests that evaluate receptive and expressive language. There are a total of 76 items in each form. In one part of these items, it is requested to show or describe a picture and, in another part, it is required to follow verbal instructions and to answer the questions verbally. The test is marked as 1 point if the child meets the passing criterion stated next to the item, and 0 point incorrectly or “not passed” if he does not. In each sub-form, the items that the child gave the correct answer by considering the minimum and the maximum point are counted and the total score is obtained. The verbal language composite score is obtained by converting the standard scores of the receptive language and expressive language subtests into oral language composite language scores. This composite score gives good information about the child's general verbal language. These results show that the test is quite successful in distinguishing children with normal language development from children with language impairment [13].

### Test of early literacy (TEL/ EROT)

Turkish validity and reliability of the EROT (Test of Early Literacy, TEL) test was performed by Kargin et al. EROT test consists of three main booklets. These are vocabulary; phonological awareness; the knowledge of alphabet and listening comprehension booklet. The first part consists of four subtests. Sub-tests of this section are; receptive and expressive language, general naming and knowledge of function. The receptive-expressive language sub-test consists of 1 sample item and 15 questions. General naming and function information consists of 1 sample item and 10 questions. The second part is composed of four sub-tests in a similar way as phonological awareness; rhyme awareness, matching according to the first voice, matching according to the last voice, dividing the sentence into words. The subtests in this section consist of 2 samples and 4 question items. The third chapter is the alphabet knowledge and listening comprehension booklet. In this part letters in receptive and expressive language is asked. In the listening comprehension section, a short story is told and 6 questions are asked to the participant. The researcher asks the children questions according to the instructions in the test battery [17]. The EROT test, consisting of seven different subtests, was performed in a quiet room in the study. The evaluation period lasted approximately 30 min. When children were bored during the test or were distracted, 10 min of breaks were given and the test was administered as two sessions.

### Data analysis

Research data were analysed with SPSS 25.0 package program. The data were analysed by the Shapiro Wilk analysis to see if it conforms to normal distribution. HA and NH children were analysed by Mann Whitney U test to see whether the data differs from school readiness test EROT. Spearman correlation test was done in order to find whether there was a relation between EROT and the study’s independent variables. Logistic regression analysis was utilized for vocabulary knowledge, phonological awareness and listening comprehension.

## Results

The study included 20 HA children aged between 5 and 6 years and 20 NH children matched by age and gender. Table 1 provides information on the demographic characteristics of children.

**Table 1:** Demographic characteristics of HA and NH children

Variables	HA		NH	
	N	%	n	%
**Gender**				
Boy	10	50.0	10	50,0
Girl	10	50.0	10	50,0
**Type of School**				
Kindergarten and Special Education	20	100.0	-	-
Kindergarten	-	-	20	100,0
**Age of Diagnosis**				
New-born Hearing Screen	13	65.0	-	-
6-12 months	3	15.0	-	-
12–18 months	2	10.0	-	-
18–24 months	1	5.0	-	-
25 months and older	1	5.0		
**Age of Hearing Aid**				
0–6 months	5	25.0	-	-
6–12 months	4	20.0	-	-
12–18 months	5	25.0	-	-
18 -24 months	4	20.0	-	-
25 month and older	2	10.0	-	-
**Degree of Hearing Loss**				
Mild	5	25.0	-	-
Moderate	6	30.0	-	-
Moderate-to- Severe	7	35.0	-	-
Severe	2	10.0	-	-
**Mother Education**				
Primary education	8	40.0	3	15,0
Secondary education	7	35.0	6	30,0
High School	5	25.0	11	55,0
**Father Education**				
Primary education	7	35.0	3	15,0
Secondary education	8	40.0	7	35,0
High School	5	25.0	10	50,0
**Mother occupation**				
Working	17	85.0	9	45,0
Housewife	3	15.0	11	55,0
**Father occupation**				
Self-employment	12	60.0	8	40,0
Government official	5	25.0	8	40,0
Worker	3	15.0	4	20,0

The participants with HA had similar pure tone threshold (PTT) means of hearing loss based on their degree of hearing loss. Participants with mild hearing loss had a PTT means of 38 dB HL, those with moderate hearing loss had a PTT means of 50 dB HL, those with moderate-to-severe hearing loss had a PTT means of 65 dB HL, and those with severe hearing loss had a PTT means of 82 dB HL.

In Table 2 it is underlined that 2 (10%) of the children with hearing loss has an above average receptive language and only 1 (5%) of the child with hearing loss has above average expressive language. On the other hand, 12 (60%) of normal hearing children have scored above average in receptive and 2 (10%) in expressive language. 15 (75%) of the children in the research group have scored average in receptive language skills and 12 (60%) in expressive language skills. In NH group 8 (%40) of the children have graded average in receptive and 18 (90%) in expressive language. Even though there seems to be no child in normal hearing group in below average grade in TELD-3 test, there are 3 (15%) children in HA group who have scored below average in receptive language skills and 7 (35%) in expressive language skills.

**Table 2:** TELD-3 receptive and expressive language impairment grade of children in the study group

Receptive Language Impairment Grade	Group	N	%
Above average	HA	2	10.0
	NH	12	60.0
Average	HA	15	75.0
	NH	8	40.0
Below average	HA	3	15.0
	NH	-	-
Expressive Language Impairment Grade			
Above average	HA	1	5.0
	NH	2	10.0
Average	HA	12	60.0
	NH	18	90.0
Below average	HA	7	35.0
	NH	-	-

The receptive language of the two groups in TELD-3 test (U = 48.00, *p* < 0.05), the expressive language (U = 51, *p* < 0.05), showed that there was a significant difference between the HA and NH groups (Table 3). Therefore, there seems to be significant difference between the recipient and expressive language test results of children with HA and NH children.

**Table 3:** Mann–Whitney U-test results in TELD-3 of HA group and NH group

	Group	N	Mean Rank	Sum of Ranks	U	p
Receptive Language	HA	20	12.90	258.0	48.000	.000
	NH	20	28.10	562.0		
Expressive Language	HA	20	13.05	261.0	51.000	.000
	NH	20	27.95	559.0		

For the second sub-objective of the study, the results of the analysis for the question ‘Is there any difference between the early literacy test measurements of vocabulary knowledge, phonological awareness, letter knowledge and listening comprehension of children with HA and NH?’ are given in Table 4.

**Table 4:** Mann–Whitney U-test results in EROT vocabulary knowledge, phonological awareness, letter knowledge and listening comprehension of HA group and NH group

	Group	N	Mean Rank	Sum of Ranks	U	p
EROT Vocabulary Knowledge	HA	20	14.15	283.00	73.000	
	NH	20	26.85	537.00		.000
EROT Phonological Awareness	HA	20	18.78	375.50	165.500	
	NH	20	22.23	444.50		.355
EROT Letter Knowledge	HA	20	14.18	283.50	73.500	
	NH	20	26.83	536.50		.000
EROT Listening Comprehension	HA	20	14.18	283.50	73.500	
	NH	20	26.83	536.50		.000

The vocabulary knowledge (U = 73.000, *p* < 0.05), letter knowledge (U = 73,500, *p* < 0.05), the listening comprehension (U = 73,500, *p* < 0.05) results showed that there was a significant difference between the HA and NH groups. However, in the phonological awareness section there seems to be no significant difference between the two groups (Table 4).

EROT Receptive Vocabulary Knowledge ((U = 73,500, *p* < 0.05), EROT Expressive Vocabulary Knowledge (U = 102,000 *p* > 0.05), EROT General Naming (U = 103,000 *p* > 0.05), EROT Knowledge Function ((U = 92,000, *p* < 0.05), EROT Receptive Letter Knowledge ((U = 96,500, *p* < 0.05) and EROT Expressive Letter Knowledge ((U = 77,500, *p* < 0.05) results showed that there was a significant difference between the HA and NH groups (Table 5).

**Table 5:** Mann–Whitney U-test results in EROT vocabulary knowledge sub-tests and letter knowledge sub-tests of HA group and NH group

	Group	N	Mean Rank	Sum of Ranks	U	p
EROT Receptive Vocabulary Knowledge	HA	20	14.18	283.50	73.500	
	NH	20	26.83	536.50		.000
EROT Expressive Vocabulary Knowledge	HA	20	15.60	312.00	102.000	
	NH	20	25.40	508.00		.007
EROT General Naming	HA	20	15.65	313.00	103.500	
	NH	20	25.35	507.00		.008
EROT Knowledge Function	HA	20	15.10	302.00	92.000	
	NH	20	25.90	518.00		.003
EROT Receptive Letter Knowledge	HA	20	15.33	306.50	96.500	.004
	NH	20	25.68	513.50		
EROT Expressive Letter Knowledge	HA	20	14.38	287.50	77.500	.001
	NH	20	26.63	532.50		

Furthermore, 20 children in the hearing aid group were divided into two subgroups: the first (11 people) with mild and moderate hearing loss, and the second (9 people) with moderate-to-severe and moderate-to-severe hearing loss. The Mann–Whitney U test revealed no statistically significant difference between the TELD-3 results of the two subgroups. On the other hand, only the EROT Phonological Awareness subtest result (z = -2.523, *p* = 0.010) revealed a significant difference between the two groups. As a result, the first group with less hearing loss had a higher level of phonological awareness.

For the third sub-objective of the study, the results of the analysis for the question ‘Do the mean scores of the receptive language, expressive language test and early literacy measurements such as vocabulary knowledge, phonological awareness, and letter knowledge and listening comprehension of HA and NH children, differ by gender?’ are given in Table 6.

**Table 6:** Mann–Whitney U-test results in TELD- 3 receptive and expressive language scores and EROT vocabulary knowledge, phonological awareness, letter knowledge and listening comprehension scores by gender

	Gender	N	Mean Rank	Sum of Ranks	U	p
EROT Vocabulary Knowledge	Boy	20	20.13	402.50	192.500	
	Girl	20	20.88	417.50		.839
EROT Phonological Awareness	Boy	20	20.40	408.00	198.000	
	Girl	20	20.60	412.00		.957
EROT Letter Knowledge	Boy	20	20.38	407.50	197.500	
	Girl	20	20.63	412.50		.942
EROT Listening Comprehension	Boy	20	20.63	412.50	197.500	
	Girl	20	20.38	407.50		.944
TELD-3 Receptive Language	Boy	20	21.00	420.00	190.000	.786
	Girl	20	20.00	400.00		
TELD-3 Expressive Language	Boy	20	20.83	416.50	193.500	.860
	Girl	20	20.18	403.50		

Vocabulary Knowledge (U = 192,500, *p* > 0.05), EROT Phonological Awareness (U = 198,000, *p* > 0.05), EROT Letter Knowledge (U = 197,500, *p* > 0.05), EROT Listening Comprehension (U = 197,500, *p* > 0.05), TELD-3 Receptive Language (U = 190,000, *p* > 0.05), TELD-3 Expressive Language (U = 193,500, *p* > 0.05) results show no significant difference according to gender (Table 6).

There is significant moderate positive correlation between age and EROT vocabulary knowledge and listening comprehension. However, there is no significant relationship between the age, gender and receptive language, expressive language, phonological awareness, and listening comprehension of the children with HA. Moreover, no significant relationship found between gender and vocabulary knowledge (Table 7).

**Table 7:** Correlation between gender, age, receptive language, expressive language scores and EROT subtests vocabulary knowledge, phonological awareness and listening comprehension

Group	r p	EROT Vocabulary Knowledge	EROT Phonological Awareness	EROT Knowledge Letter	EROT Listening Comprehension
Gender	r p	-.052	-.026	.167	.027
		.827	.913	.481	.912
Age	r p	.541	.378	.187	.518
		.014	.100	.430	.019
TELD-3 Receptive Language	r p	.325	.324	.103	.313
		.162	.163	.665	.179
TELD-3 Expressive	r	.411	.238	.022	.351
Language	p	.071	.313	.927	.129

The group of predictor variables (vocabulary knowledge, phonological awareness, letter knowledge and listening comprehension scores) with HA children results of the logistic regression analysis made for the prediction are given in Table 8.

**Table 8:** Logistic regression of vocabulary knowledge, phonological awareness and listening comprehension results for distribution according to analysis

	B	p	Odds Ratio	%95 CI
EROT Vocabulary Knowledge	.216	.035	1.241	1.015–1.516
EROT Phonological Awareness	-.200	.084	.818	.652–1.027
EROT Letter Knowledge	.140	.407	1. 151	.826–1.602
EROT Listening Comprehension	.768	.131	2.156	.796–5.836
Constant	-8.597	.005	.000	

Four subtests together and significantly did not predict in which group HA and NH children are included (*p* > 0.05). According to the analysis results, the correct classification rate in the NH group is 90%, while the correct classification rate of the measurement points in the group with children with HA is 85%. The rate of correctly classifying both groups of these scores is 87.5%. Vocabulary knowledge, phonological awareness, letter knowledge, and listening comprehension scores among the EROT subtests did not predict the HA group or the NH group (Table 8).

## Discussion

In this study no child in the normal hearing group appears to have scored below average in the TELD-3 test, there are %15 children in the HA group who have scored below average in receptive language skills and %35 in expressive language skills. In this study there appears to be a significant difference in the receptive and expressive language test results of HA and NH children. In a research made by Werfel, on receptive and expressive language tests, children with hearing loss performed low than children with normal hearing [35].

In this study, subtests of EROT, vocabulary knowledge (receptive vocabulary knowledge, expressive vocabulary knowledge and general naming, knowledge function), revealed a significant difference between the HA and NH groups. As Hirsch has underlined, the basis of understanding the language is vocabulary [14], in its most general definition is expressed as all words that children understand, know the meaning and use appropriately [23]. Given that vocabulary knowledge grows most rapidly during the early childhood era, which spans the ages of 0 to 6, it is critical to begin efforts to enhance children's vocabulary during this time to ensure potential reading success. Because it is stressed that vocabulary is a necessary ability for a child to correctly interpret the words he or she has analysed during the reading process [18, 19]. Nicholas and Geers [26] emphasized that children with hearing loss had a lower vocabulary than expected, which supported the findings of this study. In addition Kyle and Harris [21] emphasize the importance of language skills in hearing-impaired children's reading.

Moreover, in our study with subtests of EROT letter knowledge (receptive letter knowledge and expressive letter knowledge), study group was found below and there were significant differences between the groups. When research on the relationship between letter knowledge and reading are examined, it is found that children who start first grade with letter knowledge learn decoding skills faster and do better in reading [15, 17, 34]. The relationship between letter awareness at the beginning of first grade and decoding skills in the middle and at the end of first grade was explored in a study conducted by Sigmundsson et al. [32] on the subject, which included 356 children aged 5–6. According to the researchers, children who began first grade already knowing letters learned to decode faster and performed better. Easterbrooks et al. [10] states in his study that children with hard of hearing between 9 and 14 years of age showed high performance in matching letters, supporting the importance of education in letter knowledge.

In this study there was no statistically significant difference between groups in phonological awareness. When studies in the literature are examined, it can be seen in the study of Kyle and Harris that even though hearing impaired individuals can be aware of the rhymes of words, on the other hand they cannot catch up with their hearing peers even older students in their academic lives (rhyme awareness) are generally more incorrect and slower in reading [20]. In another study, the findings revealed that children with hearing loss lagged behind their peers with normal hearing in word/sentence reading fluency and the majority of the phonological awareness section [37] The difference between the literature and this study results is thought to be due to the fact that children in the study group with HA have received phonological awareness training in special education programs.

In this study when the two groups were compared based on their EROT test comprehension ratings, there was a substantial difference between them. Similar to the results of the study Yoshinaga-Itano et al. [36] have shown that people with hearing loss have more difficulty understanding stories than people who are naturally hearing. Geers [11] found that children with hearing loss who started primary school performed below grade level in terms of vocabulary knowledge and listening comprehension. Lowenstein and Nittrouer, consider that one explanation for the disparity in language and academic performance is that school language demands rise with grade level, outstripping the language abilities of children with HL [27]. Therefore, the value of developing listening comprehension skills before starting school becomes clear.

In this study Vocabulary Knowledge, EROT Phonological Awareness, EROT Letter Knowledge, EROT Listening Comprehension, TELD-3 Receptive Language, TELD-3 Expressive Language results show no significant difference according to gender.

Studies in the literature have obtained different results regarding comparison between genders. Duchesne et al. [9] did not find any correlations between the outcomes in certain language domains and predicative variables like age at implantation, gender, implant type, or educational setting. In literature, girls showed a little advantage over boys in language development in research with normal hearing children [22] as well as in studies with children using CIs [12]. Pistav Akmese et al. [30] evaluated the early literacy skills of children with cochlear implants attending a kindergarten and/or special education center with EROT. They did not find a significant difference between genders in terms of EROT subtests.

According to our study, there is a somewhat significant correlation between age and EROT listening comprehension and vocabulary knowledge. However, the children with HA's receptive language, expressive language, phonological awareness, and listening comprehension do not significantly differ by age or gender. Boons et al. [4] found that as children got older they had a significantly larger chance to be in the good performers group. With the exception of syntax, chronological age was a significant predictor of performance in all language areas. This suggests that as children with CIs become older, the chance of them having successful language outcomes rises, suggesting that they are catching up. The results of a prior study [3] showed that there was no difference in language scores at 1, 2, and 3 years following implantation. The likelihood of successful language results has increased due to the prolonged exposure with the CI and cognitive development.

Finally, the EROT subtest scores for vocabulary knowledge, phonological awareness, letter knowledge, and listening comprehension scores together significantly did not predict in which group HA and NH children are included. In Pistav Akmese and Acarlar [29] using narrative to investigate language skills of children who are deaf and with hard of hearing study, it was found that total number of words (TNW) scores predict the group with CI and number of different words (NDW) scores predict the group with NH among the language sample measurements in stories. We think that the reason why we obtained different results from Pistav Akmese and Acarlar [29] study may be due to the fact that EROT includes subtests other than language (phonological awareness, letter knowledge).

As a result, there appears to be a significant difference in the receptive and expressive language test results of HA and NH children. The results of the vocabulary knowledge, letter knowledge, and listening comprehension tests revealed a significant difference between the HA and NH groups. However, there appears to be no significant difference between the two groups in the phonological awareness section.

## Conclusion

Despite recent improvements in language growth by children with hearing loss (HL) as a result of enhanced auditory prostheses and earlier intervention, these children continue to struggle academically at higher grade levels. There is a clear connection between early literacy skills and reading and children's academic achievement, according to literature [31].

The findings of this study highlight the importance of supporting early literacy skills, which are prerequisite skills for reading and writing skills, in children who receive both mainstreaming education and special education in the risk group and/or continue their education in kindergarten.

## Data Availability

The datasets during and/or analysed during the current study available from the corresponding author on reasonable request.
